# Expression of ApoE and Spp1 in the cochlea and auditory cortex of age-related hearing loss mice

**DOI:** 10.1016/j.bjorl.2025.101674

**Published:** 2025-07-09

**Authors:** Yingxue Yuan, Junhong Zhang, Jingyi Zhao, Xiru Zhang, Zhixin Cao

**Affiliations:** a Shandong Provincial Hospital Affiliated to Shandong First Medical University, Department of Pathology, Shandong, China; b Shandong Provincial Hospital Affiliated to Shandong First Medical University, Department of Otolaryngology-Head and Neck Surgery, Shandong, China

**Keywords:** Age-related hearing loss, ApoE, Spp1, Spiral ganglion neuron, Proteomics

## Abstract

**Objective:**

Screen for differential proteins in the cochlea of mice associated with Age-Related Hearing Loss (ARHL), analyze and validate the expression of specific differential proteins and genes in the cochlea and auditory cortex of ARHL mice, and preliminarily explore their potential mechanisms of action.

**Methods:**

ABR (Auditory Brainstem Response) hearing tests were conducted to select 15-month-old C57BL/6 mice with significantly decreased hearing as the experimental group and 2-month-old mice with normal hearing as the control group. Cochleae were dissected, and unlabeled quantitative proteomics was employed to identify and analyze differentially expressed proteins in the inner ear of the two groups of mice. Key node proteins were selected via the STRING database and Cytoscape analysis. The expression of two selected proteins, ApoE and Spp1, in the cochlea was detected using qRT-PCR, Western blot, and immunofluorescence techniques, and their expression in the auditory cortex of the brain was further explored.

**Results:**

Label-free quantitative proteomics identified 115 differentially expressed proteins in the cochlea of 15-month-old ARHL mice compared to 2-month-old hearing-normal mice, including 42 upregulated and 73 downregulated proteins. GO and KEGG enrichment analyses revealed significant enrichment of differentially expressed proteins in functions and signaling pathways associated with neurodegenerative diseases and neurotransmission. Protein-Protein Interaction (PPI) analysis using the STRING database and Cytoscape selection identified ApoE and Spp1 as key hub proteins. Validation via qRT-PCR, Western blot, and immunofluorescence demonstrated that both ApoE and Spp1 were highly expressed in the cochlea and auditory cortex of the ARHL mice compare to 2-month-old hearing-normal mice.

**Conclusion:**

ApoE and Spp1 are upregulated in the cochlea of ARHL mice, particularly in spiral ganglion neurons, and in the auditory cortex, suggesting their potential involvement in the pathogenesis and progression of ARHL through the modulation of auditory neural conduction systems.

**Level of evidence:**

Level 2.

## Highlights

• Differential expression proteins in presbycusis mice identified via proteomics.
• ApoE and Spp1 upregulated in the cochlea and auditory cortex of presbycusis mice.
• ApoE and Spp1 affect auditory neural transmission, contributing to presbycusis onset.

## Introduction

Aging is becoming an increasingly pressing issue in contemporary society, and ARHL has emerged as a significant health and social concern. ARHL is typically categorized as Sensorineural Hearing Loss (SNHL), characterized primarily by a decline in the auditory perception of high-frequency sounds.^1^ Globally, it is estimated that over 400 million individuals suffer from varying degrees of hearing impairment, with the majority of these cases attributed to ARHL.^2^ ARHL can lead to serious consequences such as cognitive decline, psychological health issues, and social isolation.^3^, ^4^ The pathogenesis of ARHL involves multiple interacting factors, including genetics, aging, noise exposure, ototoxic drugs, oxidative stress, inflammation, and diet.^5^, ^6^, ^7^, ^8^ Pathological changes in ARHL often encompass the loss of cochlear hair cells, Spiral Ganglion Neuron (SGN) degeneration, atrophy of the stria vascularis, and neurodegenerative changes in the nervous system.^9^, ^10^ Recent research has gradually unveiled the underlying complex mechanisms, particularly the functional alterations within the nervous system. Studies have indicated that ARHL may lead to a decline in speech recognition ability, indicating that pathological changes might also occur in the central nervous system, especially in terms of abnormalities in the number of central neurons and synaptic information transmission.^11^, ^12^, ^13^ Against this backdrop, we employed label-free quantitative proteomics (4DLabel-free) to screen for differentially proteins in ARHL, focusing on the enrichment of proteins related to neurodegeneration, and to validate specific differential proteins. We aim to explore their expression in aged cochlear tissue and the auditory central nervous system, in hopes of providing new scientific insights and potential therapeutic targets for the prevention and treatment of ARHL.

## Methods

### Animals and grouping

C57BL/6 mice aged 2-months and 15-months were obtained from Jinan Pengyue Laboratory Animal Breeding Co., Ltd. All experiments were approved by the Animal Protection Committee of the Shandong Provincial Hospital affiliated to Shandong First Medical University (approval number: No. 2024-005) and conducted using auditory brainstem response (ABR) audiometry. The control group consisted of six 2month-old mice with normal hearing, while the experimental group consisted of six 15month-old mice with hearing loss. Each experiment was repeated at least three times.

### ABR testing

Mice were anesthetized with intraperitoneal injection of pentobarbital sodium at a dosage of 10 g/L (0.3 mL/100 g body weight). Following successful anesthesia, recording electrodes were implanted subcutaneously at the midpoint below the line connecting the two ears on the top of the skull, while two reference electrodes were inserted subcutaneously behind the ears, and a ground electrode was placed subcutaneously on the back. Short sounds (clicks) were used as the auditory stimuli. The stimulus intensity began at 90 dB and was decremented in 10 dB SPL intervals, with the lowest intensity at which Wave II could be discerned recorded as the hearing threshold. Test frequencies included click, 4, 8, 16, 24, and 32 kHz. ABRs were recorded using a TDT system of hardware and software (Tucker-Davis Technologies, Alachua, FL, USA). Throughout the procedure, mice were maintained at a stable body temperature and ensured comfort.

### Label-free quantitative proteomics analysis

Mice were euthanized by cervical dislocation. The skull was opened along the midline of the sagittal suture using fine surgical scissors, and the temporal bones were carefully excised. The cochlea was extracted using fine forceps, excess tissue was removed under a dissection microscope, and the samples were rinsed with PBS before being stored at −80 °C for further use.

Samples were ground in liquid nitrogen and precipitated with TCA/acetone (1:9) to obtain a dry powder. The powder was mixed with SDT lysis buffer, sonicated, and centrifuged to collect the supernatant. The samples underwent reduction with DTT and alkylation, followed by ultrafiltration with UA buffer and washing with NH_4_HCO_3_, before being enzymatically digested with trypsin. Peptides were desalted, re-suspended, and quantified by OD280 measurement. Mass spectrometry analysis was performed using a timsTOF Pro mass spectrometer in the PASEF mode.

The proteomic data were subjected to label-free quantification (LFQ) using the mass spectrometry database search software MaxQuant (version 1.6.14.0), the protein database UniProt (available at: http://www.uniprot.org), and the LFQ algorithm integrated within the MaxQuant software. Gene Ontology (GO) terms were obtained using Blast2GO, and pathway information involving the target protein was obtained using the KOALA (KEGG Orthology And Links Annotation) software. Enrichment analysis of GO or Kyoto Encyclopedia of Genes and Genomes (KEGG) pathway annotations was performed for the target protein set. Differentially expressed proteins were imported into the STRING 12.0 database to construct a Protein-Protein Interaction (PPI) network, which was visualized using Cytoscape software (version 3.9.1).

### Paraffin fluorescent staining

Cochlea and auditory cortex tissues were dissected and fixed with a 4% paraformaldehyde solution, with decalcification required for the cochlea. Subsequently, the tissues underwent dehydration, clarification, wax immersion, and embedding. Tissue sections with a thickness of 4 μm were prepared using a microtome. The sections were incubated with 1% Triton X-100 at room temperature for 10 min and then blocked with 5% bovine serum albumin (BSA). Primary antibodies were incubated overnight at 4 °C: anti-ApoE antibody (1:3000, ab183597, Abcam, UK), anti-Spp1 antibody (1:5000, 88742s, CST, US), and anti-Tuj1 antibody (1:500, 801201, BioLegend, US). After rewarming to room temperature for 1 h, the sections were washed with PBS and incubated with fluorescent secondary antibodies in the dark. Images were captured using a laser scanning confocal microscope.

### Protein extraction and Western blot

The cochlea and auditory cortex tissues were rapidly collected and lysed in RIPA buffer (R0020, Solarbio, CN) containing a protease inhibitor mixture. Protein concentrations were determined using the BCA Protein Assay Kit. Lysates were separated on a 10% SDS-PAGE gel and transferred to a PVDF membrane (ISEQ00010, Merck Millipore, CN). After blocking with milk, the membrane was incubated with primary antibodies: anti-β-actin (1:2000, TA-09, ZSGB-BIO, CN), anti-ApoE antibody (1:2000, ab183597, Abcam, UK), and anti-Spp1 antibody (1:2000, 88742s, CST, US) overnight at 4 °C. Following a 1-h incubation with secondary antibodies and DAPI (d9542, Sigma-Aldrich, US), immunoblots were detected using an ECL reagent kit (Merck Millipore).

### RNA extraction and qRT-PCR

Rapid collection of cochlear and auditory cortex tissues was performed, followed by extraction of total RNA using Trizol Reagent (Invitrogen, USA). Total RNA was reverse transcribed into cDNA using the Evo M-MLV Reverse Transcription Premix Kit (AG11728, AG, CN). Relative expression levels were calculated using the 2^−ΔΔCt^ method. Primers were synthesized by Suzhou Jinweizhi Biotechnology Co., Ltd. Primer sequences are listed in Table 1. Expression of β-actin was used as an internal control.

**Table 1:** Primer sequences.

Gene	Primer sequence (5’‒3’)
ApoE - Forward	GAGTGGCAAAGCAACCAACC
ApoE -Reverse	CAGTGCCGTCAGTTCTTGTG
Spp1 - Forward	CACATGAAGAGCGGTGAGTCT
Spp1 - Reverse	CCCTTTCCGTTGTTGTCCTG
β-actin - Forward	GGCTGTATTCCCCTCCATCG
β-actin - Reverse	CCAGTTGGTAACAATGCCATGT

### Statistical analysis

GraphPad Prism 10 software was used for plotting and statistical analysis. The data are presented as mean ± standard deviation (x ± s). Intergroup comparisons were performed using either the two-independent samples *t*-test or the Kruskal–Wallis rank sum test; p < 0.05 was considered statistically significant.

## Results

### ABR testing

**Fig. 1:**
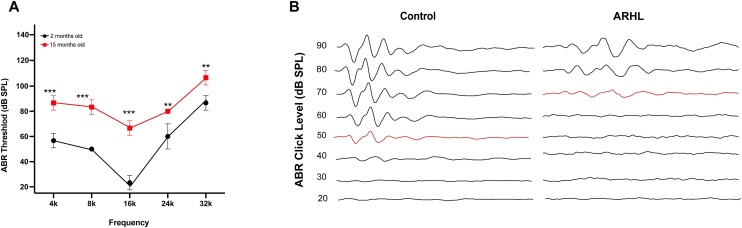
Auditory Testing. (A) ABR thresholds in 2-month-old and 15-month-old groups. (B) ABR waveforms in 2-month-old and 15-month-old groups. (** p < 0.01, *** p < 0.001, n = 3).

We assessed the auditory thresholds in 2-month-old and 15-month-old mice. ABR testing revealed that the auditory thresholds of 15-month-old mice were significantly higher than those of 2-month-old mice, particularly in the high-frequency range. The hearing thresholds of aged mice were markedly increased, indicating a more pronounced loss of high-frequency hearing. Notably, at higher sound pressure levels, the responses of the ARHL mice were significantly diminished, indicating a substantial decrease in auditory sensitivity under broadband stimulation. Specifically, at a sound pressure level of 50 dB, the 2-month-old mice could clearly exhibit auditory responses, whereas 15-month-old mice required an increase to 70 dB to elicit a response, further supporting the notion of ARHL in the elderly mice (Fig. 1).

### Identification of differentially expressed proteins in the cochlear tissue of ARHL mice

**Fig. 2:**
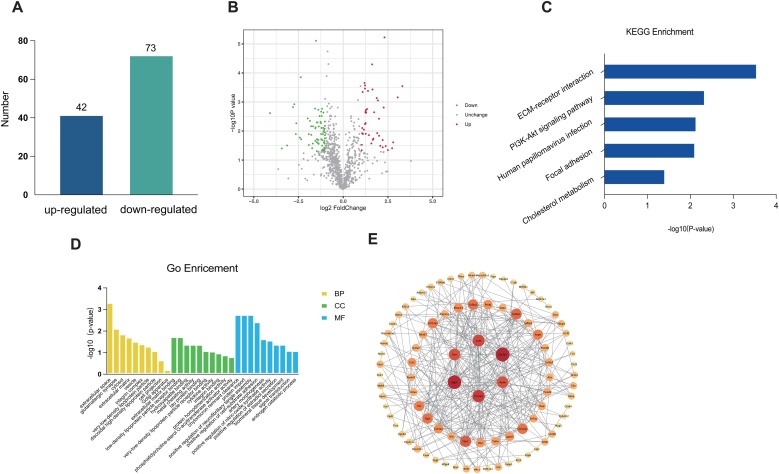
Identification and Analysis of Differentially expressed Proteins in the Cochlea of ARHL Mice. (A) The bar chart represents the number of differentially expressed proteins, with 42 proteins upregulated and 73 proteins downregulated. (B) The volcano plot of differential proteins, with red representing upregulation, green representing downregulation, and gray indicating proteins with no significant change in expression. (C) GO enrichment analysis of differential proteins, categorized into BP, CC, and MF. (D) KEGG enrichment analysis of differential proteins. (E) The PPI network diagram, where each node represents a protein and the edges between nodes represent protein-protein interactions. The greater the number of edges and the deeper the red color, the stronger the interaction.

To identify proteins associated with ARHL, we conducted a proteomic analysis of cochlear tissues from 2-month-old and 15-month-old mice. In the analysis of significant differences in the quantitative results, proteins were first filtered for statistical analysis if at least half of the replicate experimental data within the sample group were non-zero, and those with an expression fold-change greater than 2 (upregulated or downregulated) and a *t*-test p-value less than 0.05 were considered differentially expressed. The analysis revealed that there were 42 upregulated proteins and 73 downregulated proteins in the cochlear tissue of ARHL mice (Fig. 2A). All points on the volcano plot represent differential proteins between the ARHL group and the control group, with red nodes indicating upregulated proteins expression and green representing downregulated proteins (Fig. 2B).

### Bioinformatics analysis of differentially expressed proteins

We performed GO enrichment analysis and KEGG pathway analysis on significantly differentially expressed proteins to identify GO-enriched pathways and KEGG-enriched pathways associated with two proteins of interest, ApoE and Spp1. The GO enrichment analysis revealed that these two proteins are enriched in a broad range of Biological Processes (BP), potentially modulating the assembly of neurofibrillary tangles and the activity of nitric oxide synthase, thereby influencing neuronal function and the progression of related neurodegenerative diseases. They are also enriched in Molecular Functions (MF) terms related to the function, development of neural cells, and neurodegenerative diseases, such as tau protein binding, cytokine activity, and integrin binding. The most abundant Cellular Component (CC) terms involve signal transduction between neurons, particularly glutamatergic synapses. KEGG pathway enrichment identified these two differential proteins as being associated with extracellular matrix regulation, the PI3K-Akt signaling pathway, cell adhesion, and cholesterol metabolism (Fig. 2C‒D). After importing the differential proteins into the STRING 12.0 database to construct a Protein-Protein Interaction (PPI) network, we analyzed the network using Cytoscape software, setting colors and circle sizes based on the degree, which are directly proportional to the degree values. The PPI analysis revealed that ApoE and Spp1 are highly connected proteins and may serve as potential biological markers for ARHL (Fig. 2E).

### Expression of ApoE and Spp1 in mice cochlear tissues

**Fig. 3:**
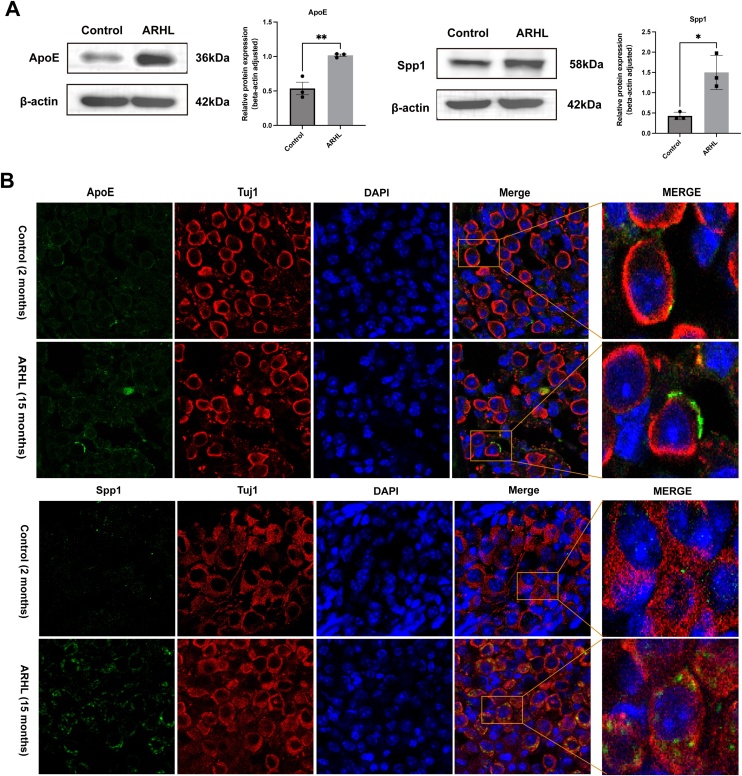
Expression of ApoE and Spp1 in cochlear tissue in ARHL and the control groups. (A) Protein Content and Statistical Graphs. (B) Paraffin Section Immunofluorescence Images of ApoE and Spp1 (green), Tuj1 (red), and DAPI (blue). (* p < 0.05, ** p < 0.01, *** p < 0.001, **** p < 0.0001, n = 3).

In this study, we systematically examined the expression of ApoE and Spp1 in the cochlear tissues of two groups of mice. Western blot analysis revealed that the protein levels of ApoE and Spp1 were significantly increased in the cochlear tissues of the ARHL group (* p < 0.05, ** p < 0.01, n = 3) (Fig. 3A). Immunofluorescence staining demonstrated that, compared to the control group, the fluorescence intensity of ApoE and Spp1 in the SGN area of the cochlea from ARHL mice was increased (Fig. 3B). These findings are consistent with the expression trends observed in our proteomic analysis.

### Expression of ApoE and Spp1 in the auditory cortex of mice

Having observed an increased expression trend of ApoE and Spp1 in the cochlear SGN, we were particularly interested in investigating their expression in the auditory cortex, which is also a key part of the auditory neural conduction system. Therefore, we examined the expression of ApoE and Spp1 in the auditory cortex of two groups of mice.

**Fig. 4:**
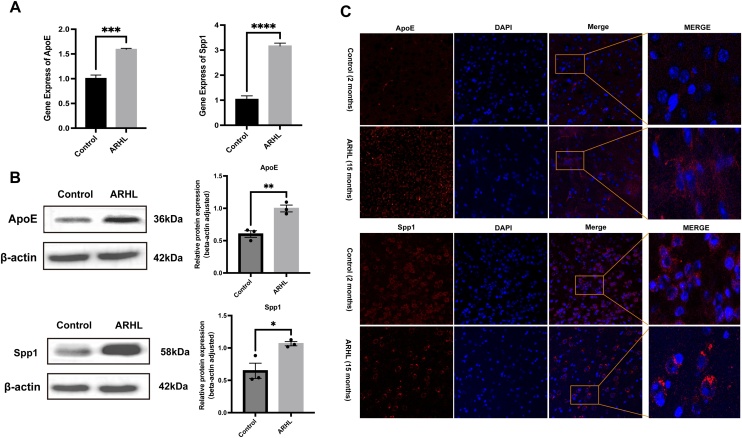
Expression of ApoE and Spp1 in the auditory cortex tissue in ARHL and the control groups. (A) mRNA expression levels of ApoE and Spp1. (B) Protein Content and Statistical Graphs. (C) Paraffin Section Immunofluorescence Images of ApoE and Spp1 (red) and DAPI (blue). (* p < 0.05, ** p < 0.01, *** p < 0.001, **** p < 0.0001, n = 3).

Through qRT-PCR, Western blot, and immunofluorescence techniques, we were excited to find the following: qRT-PCR analysis revealed a significant increase in mRNA expression of ApoE and Spp1 in the auditory cortex of the ARHL mice (*** p < 0.001, **** p < 0.0001, n = 3) (Fig. 4A). Western blot results showed elevated protein levels of ApoE and Spp1 in the auditory cortex tissues of the ARHL group (* p < 0.05, ** p < 0.01, n = 3) (Fig. 4B). Immunofluorescence indicated that the fluorescence intensity of ApoE and Spp1 in the auditory cortex of ARHL mice was higher compared to the control group (Fig. 4C).

## Discussion

ARHL, a prevalent auditory disorder associated with aging, predominantly manifests as SNHL. Beyond mere degeneration of auditory organs, ARHL involves intricate neurophysiological mechanisms. An increasing body of research has established a close relationship between ARHL and the degeneration of neural conduction pathways.^11^, ^14^ The auditory neural pathway comprises sequential structures that relay sound from the external ear to the brain. Hair cells in the cochlea detect sound and transduce mechanical vibrations into electrical signals. These electrical signals are then captured by SGNs, which transmit the signals via the auditory nerve to the cochlear nucleus in the brainstem, then through the lateral lemniscus, and ultimately via the internal capsule to the auditory cortex in the temporal lobe, where sound undergoes high-level processing such as sound recognition, comprehension, and memory.^15^ The entire neural pathway forms a complex network, comprising multiple neural structures and fibers that work in coordination to transmit auditory information from the ear to the brain. SGNs and the auditory cortex, as key neural components in the transmission of auditory signals from the cochlea to the brain, are critically involved in the perception of auditory signals. Their damage and degeneration can severely affect the perception of these signals.

With the advancement of high-throughput technologies, multiple proteins have been detected and utilized for the prediction of potential biomarkers for ARHL through proteomic analysis and bioinformatics tools. In this study, we employed LFQ proteomics to identify and analyze differentially expressed proteins in the cochlea of ARHL mice, and used bioinformatics analysis to recognize key biomarkers and relevant pathways associated with ARHL. GO and KEGG enrichment analyses revealed that differentially expressed proteins were significantly enriched in pathways associated with neurodegenerative changes (e.g., glutamatergic synapse) and neural conduction (e.g., PI3K-Akt signaling pathway). Through experimental studies and bioinformatic analysis, we selected two proteins Spp1 and ApoE that were significantly elevated in the cochlear nerve tissue and brain tissue of ARHL mice. Based on literature review, we concluded that both of them may participate in ARHL by affecting nerve conduction, but may involve different pathological mechanisms and coordinate the occurrence and development of ARHL.

Apolipoprotein E (ApoE), a protein primarily involved in lipid metabolism and cholesterol transport, plays a critical role in the circulation of cholesterol and other lipids in both the bloodstream and central nervous system.^16^ The ApoE gene has three common alleles: ε2, ε3, and ε4. The ApoE ε2 and ε3 alleles are generally considered to have a protective effect on hearing,^17^ while the ε4 allele is significantly associated with an increased risk of Alzheimer's disease. Individuals carrying the ApoE ε4 allele often exhibit a higher risk of hearing loss in later life.^18^ The impact of ApoE on hearing may vary depending on the specific allele, as each allele may exert different effects. Recent studies have shown that the ApoE ε4 allele in the brain can stimulate microglial proliferation and secretion of inflammatory mediators, exacerbating neuroinflammation in the brain.^19^ Moreover, ApoE ε4 can contribute to the development and progression of neurodegenerative diseases by affecting the metabolism and clearance of amyloid-β, cellular membrane stability, and neuronal function.^20^ It is known that the inner ear contains abundant ganglion and glial components that transmit auditory signals, which are similar to the cellular components of brain tissue.^21^ A study published in Scientific Reports in 2024 showed that the ApoE ε4 allele may contribute to the development of Age-Related Hearing Loss (ARHL) by affecting the function and structure of cochlear Spiral Ganglion (SGN) cells.^22^ Therefore, the elevated expression of ApoE ε4 could influence hearing in a manner similar to its effects on brain tissue, potentially impairing auditory nerve conduction and leading to ARHL. Based on this, we hypothesize that in the aging hearing loss mouse model used in this study, the significantly increased expression of ApoE protein in cochlear tissue may indicate a more critical role of the ApoE ε4 allele. Although genotyping of ApoE alleles was not performed in this study, our findings suggest a potential association between elevated ApoE expression and the ε4 allele in ARHL. Future studies will validate this hypothesis through targeted sequencing. Additionally, the expression of ApoE in different types of hearing loss (such as presbycusis, traumatic, or ototoxic hearing loss) may vary, offering new insights for further exploration of ApoE's role in hearing loss.

In this study, we found that compared to young mice with normal hearing, the expression of ApoE protein was significantly elevated in the cochlea and auditory cortex of ARHL mice, suggesting that the ApoE gene may contribute to the progression of hearing loss by affecting the health of the auditory nervous system. Although we did not conduct a specific genotyping study of ApoE alleles, based on the above analysis, we hypothesize that the increased expression of ApoE in the auditory central nervous system may be related to the role of the ε4 allele in neurodegeneration in the brain tissue of aging mice. The exact mechanism of ApoE’s role in ARHL remains to be further elucidated, but these findings provide preliminary evidence for the involvement of ApoE in the auditory system and may offer potential targets for future therapeutic strategies.

Secreted Phosphoprotein 1 (Spp1), also known as osteopontin, is a multifunctional phosphoglycoprotein that is widely expressed across various tissues and cell types, playing significant roles in numerous biological processes, including immune regulation, cell adhesion, migration, and signal transduction.^23^, ^24^, ^25^, ^26^ Studies have shown that during the progression of AD, Spp1 promotes microglial phagocytosis of functional neuronal synapses, leading to a reduction in synaptic density and impairing neural transmission.^27^ Furthermore, Spp1 has been shown to regulate the function of SGNs, which is critical for the transmission of auditory signals from the cochlea to the brain. Overexpression or dysregulation of Spp1 may contribute to inflammation and neuronal damage associated with ARHL.^28^ It is known that oxidative stress is a significant factor in the development of ARHL, and research has found that Spp1 can reduce cochlear metabolism and oxidative stress levels in a murine model of presbycusis by regulating genes responsive to oxidative stress, suggesting that targeting Spp1 may help alleviate oxidative stress in the auditory system. Bioinformatics analysis in this study revealed that the protein expression profiles in the cochlear tissues of ARHL mice were significantly enriched in GO terms and KEGG pathways associated with inflammatory responses, such as cytokine activity, positive regulation of nitric-oxide synthase activity, focal adhesion, PI3K-Akt signaling pathway, and ECM-receptor interaction (Fig. 2C and D). While we did not directly measure the markers in ARHL mice. Future work will assess ROS levels and inflammatory cytokines (e.g., IL-6) to elucidate their mechanistic roles. In this study, we observed a significant increase in Spp1 expression in both cochlear SGNs and glial cells in the auditory cortex in ARHL mice. Considering the role of Spp1 in AD models, we hypothesize that Spp1 may be involved in the pathological processes of ARHL by inducing microglial phagocytosis of neuronal synapses and promoting oxidative stress. Considering the complex expression pattern of Spp1 in ARHL, its expression in the cochlea may vary depending on the mouse strain and the different stages of hearing loss. We will further investigate this phenomenon and the underlying mechanisms in subsequent studies.

In this study, we employed multiple databases for cross-validation and conducted enrichment analysis using the latest data from multi-source GO and KEGG databases to ensure the comprehensiveness of the results. However, as the research progresses, database information will continue to be updated. In the future, we will use experimental methods such as co-immunoprecipitation to validate the predicted interactions and refine the current analysis. At the same time, the results of this study only reflect the potential roles of ApoE and Spp1 in ARHL and do not fully account for factors such as oxidative stress and inflammation. Future research will systematically assess other influencing factors in a broader physiological and pathological context.

## Conclusion

We identified and validated key differential proteins associated with ARHL via bioinformatics analysis, with a particular focus on ApoE and Spp1. The experimental results demonstrated that ApoE and Spp1 are upregulated in the cochlea of ARHL mice, particularly in spiral ganglion neurons, and in the auditory cortex, suggesting their potential involvement in the pathogenesis and progression of ARHL through the modulation of auditory neural conduction systems.

## ORCID ID

Yingxue Yuan: 0009-0001-8795-8777

Junhong Zhang: 0009-0005-7977-1370

Jingyi Zhao: 0009-0000-1673-8198

Xiru Zhang: 0000-0001-8822-2596

## Funding

10.13039/501100001809National Natural Science Foundation of China (Grant nº 82201293), the (Grant nº 82201293); 10.13039/501100007129Natural Science Foundation of Shandong Province (Grant nº ZR2021MH369). Youth Science Foundation Cultivation Support Program of Shandong First Medical University (Grant nº 202201-061).

## Declaration of competing interest

The authors declare no conflicts of interest.
